# Draft Genome Sequence of *Avibacterium paragallinarum* Strain 221

**DOI:** 10.1128/genomeA.00290-13

**Published:** 2013-05-23

**Authors:** Fuzhou Xu, Deyuan Miao, Yu Du, Xiaoling Chen, Peijun Zhang, Huiling Sun

**Affiliations:** Beijing Key Laboratory for Prevention and Control of Infectious Diseases in Livestock and Poultry, Institute of Animal Husbandry and Veterinary Medicine, Beijing Academy of Agriculture and Forestry Sciences, Beijing, Chinaa; Bioinformatics Program, Seneca College of Applied Arts & Technology, Toronto, Canadab; BGI-Shenzhen, Shenzhen, Chinac

## Abstract

*Avibacterium paragallinarum* is the causative agent of infectious coryza. Here we report the draft genome sequence of reference strain 221 of *A. paragallinarum* serovar A. The genome is composed of 135 contigs for 2,685,568 bp with a 41% G+C content.

## GENOME ANNOUNCEMENT

*Avibacterium paragallinarum*, a member of the *Pasteurellaceae* family, is a Gram-negative bacterium which in chickens causes infectious coryza, an acute respiratory disease that has worldwide economic significance (1). This microorganism can be either nicotinamide adenine (NAD) dependent or independent in growth character (2–4). Two serotyping schemes have been widely used for *A. paragallinarum*—serovars A, B, and C for the Page serotyping scheme (5) and serogroups I, II, and III for the Kume serotyping scheme (6). Subsequent publications had reported the recognition that the three Kume serogroups correspond to the three Page serovars (7). More variants of *A. paragallinarum* have emerged through serotyping and vaccination failures (1).

One of the promising strategies for the control of infectious coryza is to study the pathogenesis of *A. paragallinarum* in chickens. However, up to now there has been little information concerning the genome sequence of *A. paragallinarum*. To investigate the genomic basis of the pathogenesis of *A. paragallinarum*, reference strain 221 of *A. paragallinarum* serovar A was used for whole-genome sequencing.

The genomic DNA of *A. paragallinarum* strain 221 was extracted and purified from a fresh bacterial culture using the DNeasy blood and tissue kit (Qiagen Corp.) according to the manufacturer’s instructions. The genomic DNA was sequenced by a whole-genome shotgun strategy using Illumina Highseq 2000. Genomic libraries of 500 bp and 6 kb were constructed, sequenced, and assembled using SOAPdenovo software with at least 100-fold coverage and generated 103 scaffolds containing 135 contigs. The largest contig was approximately 178 kb and the minimum contig was 230 bp. The genome of *A. paragallinarum* strain 221 is 2,685,568 bp in length and has an average G+C content of 41%. Automatic gene prediction was performed using Glimmer 3.0 (8). In total, 2,994 protein-encoding genes were predicted, with a coding percentage of 87.3%. The average gene length is 783 bp. Nine genomic islands were also identified by this software. In addition, the contigs were searched against the KEGG (9) and COG (Clusters of Orthologous Groups) (10) databases to annotate the gene description. Based on the analysis, functions of 2,694 genes (90% of the total of 2,994 genes) were predicted and 2,271 genes (75.9%) were assigned into 22 clusters of COG functions. Moreover, 13 predicted copies of 5S, 16S, and 23S rRNA genes and 52 predicted tRNA genes were detected using rRNAmmer (11) and tRNAscan (12) softwares, respectively.

### Nucleotide sequence accession numbers.

This whole-genome shotgun project has been deposited at DDBJ/EMBL/GenBank under the accession number AOGF00000000. The version described in this paper is the first version, AOGF01000000.
